# Targeting CK2 mediated signaling to impair/tackle SARS-CoV-2 infection: a computational biology approach

**DOI:** 10.1186/s10020-021-00424-x

**Published:** 2021-12-20

**Authors:** Jamilet Miranda, Ricardo Bringas, Jorge Fernandez-de-Cossio, Yasser Perera-Negrin

**Affiliations:** 1 Division of Informatics, Department of Bioinformatics, Center for Genetic Engineering and Biotechnology, Havana, Cuba; 2 Laboratory of Molecular Oncology, Division of Biomedical Research, Department of Pharmaceuticals, Center for Genetic Engineering and Biotechnology, Havana, Cuba; 3 China-Cuba Biotechnology Joint Innovation Center, Yongzhou Zhong Gu Biotechnology Co., Yongzhou, Hunan People’s Republic of China

**Keywords:** CIGB-300, SARS-CoV-2, Phosphoproteomics, CK2 inhibitor, Drug repurposing, Computational biology, NPM1/B23, SQSTM1, SRSF1, HSBP1

## Abstract

**Background:**

Similarities in the hijacking mechanisms used by SARS-CoV-2 and several types of cancer, suggest the repurposing of cancer drugs to treat Covid-19. CK2 kinase antagonists have been proposed for cancer treatment. A recent study in cells infected with SARS-CoV-2 found a significant CK2 kinase activity, and the use of a CK2 inhibitor showed antiviral responses. CIGB-300, originally designed as an anticancer peptide, is an antagonist of CK2 kinase activity that binds to the CK2 phospho-acceptor sites. Recent preliminary results show the antiviral activity of CIGB-300 using a surrogate model of coronavirus. Here we present a computational biology study that provides evidence, at the molecular level, of how CIGB-300 may interfere with the SARS-CoV-2 life cycle within infected human cells.

**Methods:**

Sequence analyses and data from phosphorylation studies were combined to predict infection-induced molecular mechanisms that can be interfered by CIGB-300. Next, we integrated data from multi-omics studies and data focusing on the antagonistic effect on the CK2 kinase activity of CIGB-300. A combination of network and functional enrichment analyses was used.

**Results:**

Firstly, from the SARS-CoV studies, we inferred the potential incidence of CIGB-300 in SARS-CoV-2 interference on the immune response. Afterwards, from the analysis of multiple omics data, we proposed the action of CIGB-300 from the early stages of viral infections perturbing the virus hijacking of RNA splicing machinery. We also predicted the interference of CIGB-300 in virus-host interactions that are responsible for the high infectivity and the particular immune response to SARS-CoV-2 infection. Furthermore, we provided evidence of how CIGB-300 may participate in the attenuation of phenotypes related to muscle, bleeding, coagulation and respiratory disorders.

**Conclusions:**

Our computational analysis proposes putative molecular mechanisms that support the antiviral activity of CIGB-300.

**Supplementary Information:**

The online version contains supplementary material available at 10.1186/s10020-021-00424-x.

## Introduction

The spread of severe acute respiratory syndrome coronavirus 2 (SARS-CoV-2), with increasing levels of infectivity and transmissibility, has strained health systems worldwide. Due to the need of finding effective therapeutic treatments in the shortest possible time, drug repurposing has emerged as the first option (Serafin et al. 2020; Zhou et al. 2020b). The huge amount of data generated permits the re-consideration of drugs already evaluated for other diseases, which might have advanced toxicological, preclinical and/or clinical studies.

Since the genomic sequence of SARS-CoV-2 became available in January 2020 (Zhou et al. 2020a), a diversity of techniques and laboratory models have been applied to study SARS-CoV-2 replication and infectivity.

Blanco-Mello et al. (2020) performed RNA-seq experiments from polyA RNAs isolated from infected cells. They found a reduced transcriptional response of type I/III interferon-induced genes and, concurrently, a significant increase of chemokines and IL6, by which they suggested the evaluation of FDA-approved drugs with immunomodulating properties, which could be rapidly implemented in clinical protocols.

Various mass-spectrometry studies contributed importantly to the understanding of the life cycle of SARS-CoV-2. Gordon et al. (2020) using affinity-purification mass spectrometry (AP-MS), identified the sets of human proteins that physically interact with each one of the 26 viral proteins that they had individually cloned in human cells derived from kidney, HEK-293T/17 cell line. A total of 332 physical interactions between SARS-CoV-2 and human proteins were identified. On analyzing the expressions of all interacting human proteins in 29 tissues, they identified those of the lung as having the highest expression levels. Gene Ontology (GO) enrichment analysis was performed to the set of human protein interactors for each viral protein cloned. For each one of the 26 sets, the main overrepresented biological processes included Nuclear Transport, Ribonucleoprotein Complex Biogenesis and Cellular Component Disassembly. Host proteins involved in the innate immune response were targeted by viral proteins nsp13, nsp15 and orf9b while proteins from the Nf-kB pathway were targeted by nsp13 and orf9c. The most relevant host proteins targeted by known drugs were identified from this analysis. Afterwards, they demonstrated the capacity of some of these drugs to reduce viral infectivity. The study of Gordon et al. (2020) is of outstanding relevance for the understanding of the mechanism used by SARS-CoV-2 to improve its infectivity and to avoid a strong immune response. It also provides valuable information for the repurposing of the existing drugs.

The role of kinases in the course of viral infection was addressed by Bouhaddou et al. (2020), who carried out a quantitative mass spectrometry-based phosphoproteomic study in Vero E6 cells infected by SARS-CoV-2. Casein kinase II (CK2) and p38 MAP kinases were significantly activated while mitotic kinases were shut down. A relevant role of CK2, associated with viral capsid protein N, in induced filopodia protrusions was observed during viral infection. Both CK2 and protein N were co-localized in filopodia protrusions, which were significantly longer and more branched than in control cells. The authors suggest that the N protein may control CK2 activity and regulate cytoskeleton elements in filopodia. Although the role of CK2 in viral infections is not new, it is remarkable the level of upregulation of the CK2 activity after SARS-CoV-2 infection (Bouhaddou et al. 2020). The strong antiviral activity of Silmitasertib (CX-4945), a CK2 inhibitor, suggests that this kinase is an attractive target for Covid-19 treatment. An ongoing clinical trial of CX-4945 is evaluating its clinical benefits and anti-viral activities in moderately ill COVID-19 patients (Recknor 2020).

CIGB-300 is a synthetic peptide designed to bind the phospho-acceptor motif of CK2 substrates, interfering in the phosphorylation of serine/threonine residues by CK2 (Perea et al. 2008). The safety and tolerability of the intravenously administered CIGB-300 was confirmed through its clinical use in cancer patients (Perea et al. 2015; Batista-Albuerne et al. 2018).

In a phosphoproteomic experiment in NCI-H125 cells, Perera et al. (2020) identified CK2 phospho-acceptor peptides that are significantly inhibited by CIGB-300. They found, for the first time, that CIGB-300 binds to the CK2α subunit and impairs the CK2α2β2 holoenzyme enzymatic activity. In contrast, the phosphorylation of the CK2β subunit, which contains a consensus CK2 phosphorylation motif, was not influenced by CIGB-300. Additionally, Perera et al. (2009) identified nucleophosmin (B23) as a major target of CIGB-300. Moreover, Nouri et al. (2015) further reported the binding of CIGB-300 to the B23 oligomerization domain. This interaction blocks the association of B23 to Rev and US11 proteins, two functionally homologous proteins from HIV and HSV viruses, respectively. Cells treated with CIGB-300 showed a significant reduction of virus production, suggesting that B23 is an attractive target for antiviral drugs. Lobaina and Perera (2019) also proposed the use of B23 as a potential target in antiviral therapies.

Considering this background, CIGB-300 was tested for its safety and clinical benefits in Covid-19 patients in a phase I/II clinical trial (Cruz et al. 2020). It reduced the number of pulmonary lesions among treated individuals. Additionally, CIGB-300’s antiviral effect on MDBK cells infected with bovine coronavirus (BCoV) Mebus was explored (Ramón et al. 2021). CIGB-300 inhibited the cytopathic effect and reduced viral protein accumulation in the cytoplasm. The physical interaction of CIGB-300 with the BCoV nucleocapsid protein (N) was also revealed. Through functional enrichment, it was observed that cytoskeleton reorganization and protein folding were the main biological processes disturbed.

Here we present an in silico analysis of SARS-CoV and SARS-CoV-2 viral infection. We performed a multi-omics integrative analysis of SARS-CoV-2 infection in human cell lines that combined functional enrichment and network representation. At the level of phosphorylation sites, we integrated data from four phosphoproteomic studies on SARS-Cov-2 infection (Bouhaddou et al. 2020, Hekman et al. 2020, Klann et al. 2020, Stukalov et al. 2021) and one study on CIGB-300 inhibition of kinase activity (Perera et al. 2020). We identified, at different times after viral infection, the biological processes and virus activated phosphosites that can be interfered by CIGB-300.

## Materials and methods

### Public data

Protein sequence information was downloaded from the UniprotKB database (Pundir et al. 2017) https://www.uniprot.org/.

Data sources from phosphoproteomic studies of host and viral proteins of SARS-CoV-2 infected human cell lines were downloaded from the sites listed in Table 1.

**Table 1:** SARS-CoV-2 infection: data sources of phosphoproteomic studies

Reference	Timepoints	Cell lines	Downloaded from
Bouhaddou et al. (2020)	2 h, 4 h, 8 h, 12 h and 24 h	Vero E6	http://dx.doi.org/10.17632/dpkbh2g9hy.1
Hekman et al. (2020)	1 h, 3 h, 6 h and 24 h	iAT2	https://www.ebi.ac.uk/pride/archive/projects/PXD020183
Stukalov et al. (2021)	6 h and 24 h	A549	https://www.nature.com/articles/s41586-021-03493-4#MOESM5
Klann et al. (2020)	24 h	CaCo-2	https://www.cell.com/molecular-cell/fulltext/S1097-2765(20)30549-9#supplementaryMaterial

The phosphorylation data of the CIGB-300 treatment of Perera et al. (2020) were downloaded from https://link.springer.com/article/10.1007%2Fs11010-020-03747-1#additional-information/.

Protein expression data of Bojkova et al. (2020) were downloaded from http://corona.papers.biochem2.com/.

A total of 332 human-SARS-CoV-2 protein–protein interactions (PPI) of Gordon et al. (2020) were downloaded from the Biogrid database (Stark et al. 2006) at https://thebiogrid.org/225737/publication/comparative-host-coronavirus-protein-interaction-networks-reveal-pan-viral-disease-mechanisms.html#!.

Information of CK2 Substrates was downloaded from PhosphoSitePlus at (https://www.phosphosite.org/) (Hornbeck et al. 2015).

### Data analysis

Sequence alignments were performed with the desktop version of CLUSTALX multiple sequence alignment program (Thompson et al. 1997).

Analysis of proteomic data from Bojkova et al. (2020) was carried out with MEV (Saeed et al. 2006). The SAM method (Tusher et al. 2001) for the two-class unpaired comparison was used to identify the most differentially expressed proteins as implemented in MEV. Parameter delta was set so that the estimated median number of false significant proteins would be cero.

In the case of data on phosphorylation induced by SARS-CoV-2 infection (Bouhaddou et al. 2020, Stukalov et al. 2021, Klann et al. 2020 and Hekman et al. 2020), the criteria used to choose differentially phosphorylated sites was log2 fold change ≥ 0.25 and adjusted-p-value ≤ 0.05. These were the same values proposed by Hekman et al. (2020).

The phosphorylation changes induced by the CIGB-300 treatment were extracted from Perera et al. (2020). All inhibitions reported by the authors at 10 and/or 30 min were considered.

For the Gene Set Enrichment Analysis we used GSEA version 4.1.0 for Windows (Mootha et al. 2003; Subramanian et al. 2005, 2007). Gene lists ordered by fold change values for each time point post infection were provided as input. The pre-ranked gene list option was used for databases containing REACTOME and GO biological process gene sets. MSigdb v7.2 gmt files were downloaded from: http://www.gsea-msigdb.org/gsea/downloads.jsp. The permutation-based p-value is corrected for multiple testing to produce a false-discovery rate (FDR) q-value that ranges from 0 (highly significant) to 1 (not significant). In this case, the criteria used for statistical significance was a Nominal p-value threshold of 0.05 and a False Discovery Rate (FDR) of 0.25, as recommended by the GSEA software.

The Cytoscape tool (Shannon et al. 2003, Smoot et al. 2011) was used to build and merge networks in Figs. 7 and 8.

BisoGenet Cytoscape plugin (Martin et al. 2010), available from Cytoscape Application Manager, was used to generate PPI networks.

Venn Diagrams were generated using the web application at: http://bioinformatics.psb.ugent.be/.

Functional analysis of enriched pathways and reactions was performed using Reactome Pathway Knowledgebase (Jassal et al. 2020) at: https://reactome.org/. The criteria used for selection was FDR ≤ 0.05.

GeneCodis 4.0, at https://genecodis.genyo.es/, was used for the disease enrichment analysis (Tabas-Madrid et al. 2012). Data sources for Human Phenotypes HPO and OMIM were both consulted individually and integrated. The results were obtained in the form of network clusters.

For building the 3D protein structure model we used Swiss-Model (Waterhouse et al. 2018) at https://swissmodel.expasy.org/ and for the interactive visualization of the model generated we used the Chimera desktop application (Goddard et al. 2018).

BiNGO plugin (Maere et al. 2005), available from Cytoscape Application Manager, was used to determine and visualize GO categories statistically overrepresented.

Additional statistical analysis and graphs were generated and plotted using GraphPad Prism version 5.00 software (GraphPad Software, San Diego, CA, USA).

## Results

### Inferring the potential effect of the CIGB-300 treatment on SARS-CoV-2 virus infection based on previous results

We first attempted to extrapolate findings on SARS-CoV virus infection to SARS-CoV-2. Proteins N and Orf6 are well conserved in both viruses and have been reported to play important roles in viral pathogenesis (Surjit and Lal 2008; Frieman et al. 2007). Both proteins are known to contain putative CK2 phospho-acceptor sites (Surjit et al. 2005, Klann et al. 2020). Afterwards, we ventured to anticipate the potential effect of CIGB-300 on the viral pathogenesis of SARS-CoV-2.

### CIGB-300 could alter N-protein localization and its RNA binding capacity

**Fig. 1:**
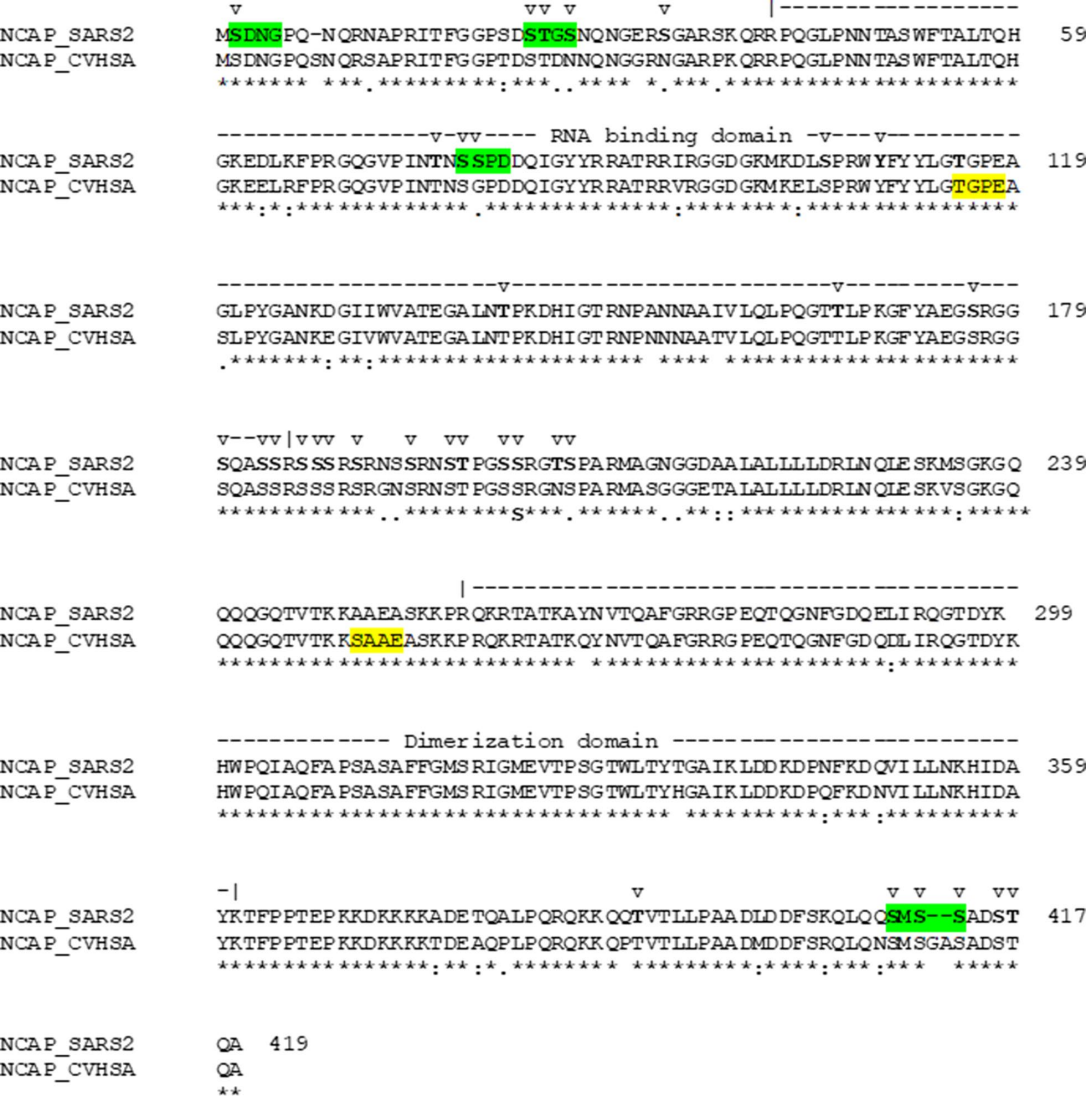
Sequence alignment of the nucleocapsid proteins of SARS-CoV-2 (NCAP_SARS2) and SARS-CoV (NCAP_CVHSA) viruses. Numbers at the right end indicate the amino acid position in the SARS-CoV-2 protein. Above the aligned sequences, the RNA-binding (residues 41–186, according to UniProt annotation) and Dimerization (residues 258–361) domains are delimited. The v-above the amino acid residues indicates the phosphorylation sites identified in the works of Davidson et al. (2020), Bouhaddou et al. (2020), Klann et al. (2020) and Hekman et al. (2020). The highlighted segments are those between a CK2 phospho-acceptor site and position + 3, those amino acids in yellow are the ones in silico predicted by Surjit et al. (2005) and in green are those experimentally validated by Davidson et al. (2020)

A sequence alignment of N protein from SARS-CoV and SARS-CoV-2 viruses shows a strong similarity (Fig. 1). N Protein consists of two structural domains (Fig. 1): the N-terminal RNA-binding domain (residues 41–186), and the C-terminal dimerization domain (residues 258–361). The rest of the protein is highly disordered (Chang et al. 2006).

Surjit et al. (2005) predicted that the SARS-CoV N-protein would be heavily phosphorylated. Thr116 and Ser251 were noted as putative phospho-acceptors for CK2 (see Fig. 1), although this has not been experimentally verified. We collected a total of 33 phosphorylation sites for the SARS-CoV-2 N protein (see Fig. 1 and Additional file 1: Table S1) from four recent mass spectrometry studies (Bouhaddou et al. 2020, Davidson et al. 2020, Hekman et al. 2020, Klann et al. 2020). Davidson et al. (2020) reported two putative CK2 sites at Ser2 and Ser78. Hekman et al. (2020) found that Ser23 and Ser410 were phosphorylated by CK2.

Bouhaddou et al. (2020) analyzed the impact of phosphorylation on the N-protein surface charges by a 3D structural model of the RNA-binding domain. These changes may modulate the function of the N-protein by regulating its RNA binding capacity. One of the phosphorylation sites responsible for these charge changes is Ser78 (see Fig. 1), a CK2 phospho-acceptor site according to Davison et al. (2020). The binding of CIGB-300 to Ser78 would then interfere with the RNA binding ability of the N-protein.

On the other hand, the N protein is reported to be mainly located in the cytoplasm (Chang et al. 2004; Surjit et al. 2005; Zhang et al. 2020). However, a localization analysis of N-expressing cells treated with four different phosphorylation inhibitors found a significant fraction of the N protein located in the nucleus of cells treated with CDK or CK2 inhibitors (Surjit et al. 2005). Additionally, in cells infected by BCoV, CIGB-300 bound to the N protein, downregulated its expression and significantly reduced the accumulation of viral proteins in the cytoplasm (Ramon et al. 2021).

Bouhaddou et al. (2020) found CDK activity to be significantly reduced by SARS-CoV-2 infection while CK2 activity is significantly increased. Consequently, the inhibition by CIGB-300 of the N protein phosphorylation sites may alter, at least in part, its cytoplasmic localization. Hence, the use of CIGB-300 in Covid-19 patients would interfere in the N protein’s role in the viral life cycle of infected cells, since its function in particle assembly occurs in the cytoplasm.

### CIGB-300 could bind to the Orf6 C-terminus and restore IFN signaling

**Fig. 2:**
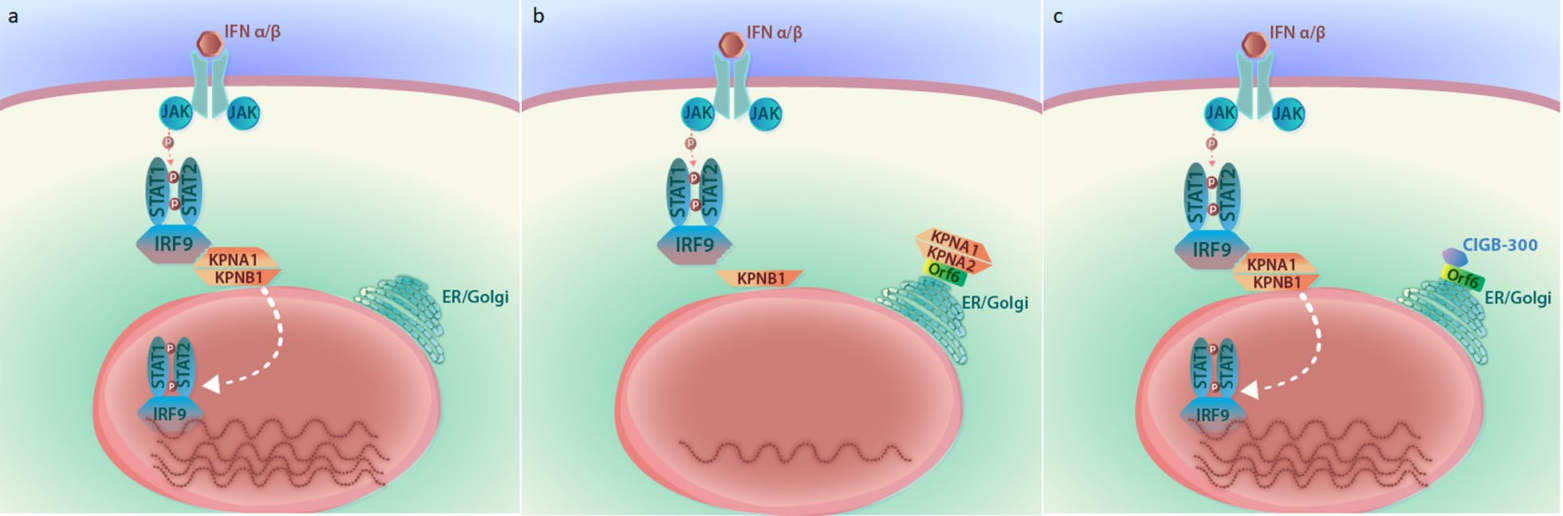
Interference of IFN signaling by Orf6 and the role of CIGB-300. **a** Interferon, on binding to the receptor, induces STAT1 phosphorylation and the formation of a complex with STAT2 and IRF9. KPNA1 binds with the complex, and KPNB1 binds to KPNA1 and thereby chaperons the complex through the nuclear pore. **b** Orf6 retains KPNA1 and KPNA2 in the ER/Golgi membrane and the transport of the STAT complex to the nucleus is interrupted. **c** CIGB-300 blocks the interaction of Orf6 with KPNA2 and the transport of the STAT complex to the nucleus is restored

An important element of the innate immune response to virus infections is the activation of antiviral genes as a consequence of interferon production. After the activation of receptors by type I interferons, STAT1 is phosphorylated and forms a complex with STAT2 and IRF9 (Schneider et al. 2014). This complex exposes a nuclear localization signal (NLS) that is bound by KPNA1, and as a last step before entering the nucleus, KPNB1 binds to KPNA1 and chaperons the complex through the nuclear pore (Fig. 2a) (Frieman et al. 2007).

Several groups have attributed an antagonistic immune response effect to the Orf6 protein (Frieman et al. 2007, Li et al. 2020b, Yuen et al. 2020). In SARS-CoV experiments, Frieman et al. (2007) reported that Orf6 interferes with the immune response of the host by antagonizing the STAT1 function. Orf6 binds to karyopherin alpha 2 (KPNA2) and retains it in the ER/Golgi membrane. KPNB1 is also retained as it binds to KPNA2. In this way, the chaperon function of KPNB1 through the nuclear pore is interfered, and STAT1 signaling is interrupted (Fig. 2b).

**Fig. 3:**
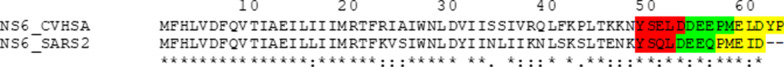
Sequence alignment of Orf6 proteins of SARS-CoV (NS6_CVHSA) and SARS-CoV-2 (NS6_SARS2) viruses. The residues highlighted in red, green and yellow are those replaced by alanine in the mutants generated by Frieman et al. (2007) and Lei et al. (2020)

Frieman et al. (2007) also found that the ten C-terminal amino acids of SARS-CoV Orf6 are responsible for KPNA2 binding. In Fig. 3 we show the residues of Orf6 involved in the SARS-CoV mutants they generated, Orf6_49-53Ala_, Orf6_54-58Ala_ and Orf6_59-63Ala_ (author’s nomenclature), by replacing amino acids 49–53, 54–58 and 59–63 with alanine, respectively. The last two mutants, Orf6_54-58Ala_ and Orf6_59-63Ala_, comprising the ten C-terminal amino acids, did not retain KPNA2, and as consequence, STAT1 function was unaffected. The first mutant Orf6_49-53Ala_ was still able to retain KPNA2. Hence, the last ten amino acids were responsible for KPNA2 binding and, as consequence, for KPNB1 recruitment.

Recently Lei et al. (2020) carried out a similar mutation study of the SARS-CoV-2 Orf6 protein. They generated three mutants; M1, M2 and M3 (author’s nomenclature); by replacing amino acids 49–52 (YSQL), 53–56 (DEEQ) and 57–61 (PMEID) by alanine, respectively (Orf6 of SARS-CoV-2 lacks the last two amino acids present in the SARS-CoV protein). As expected, they obtained similar results: mutant M1 perturbs interferon stimulation as with the wild type, while mutants M2 and M3 lack the inhibitory effect.

In Fig. 3 we show an alignment of Orf6 protein sequences from SARS-CoV and SARS-CoV-2 viruses. The region between amino acids 50–53 with the SELD sequence in the SARS-CoV protein and the SQLD sequence in SARS-CoV-2, both match the CK2 substrate motif. Additionally, this site in SARS-CoV-2 was experimentally found to be phosphorylated, and it was predicted, by computer analysis, to be a phospho-acceptor site of CK2 (Klann et al. 2020). Since Ser50 is a CK2 phospho-acceptor site, it may also be bound by CIGB-300. Mutant M2 of Lei et al. (2020) includes the Asp53 residue at position + 3 relative to Ser50, and this position is known to be important for the recognition of CK2. Therefore, we strongly suggest that the possible binding of CIGB-300 to this phospho-acceptor motif could interfere in the interaction of Orf6 C-terminus with KPNA2; thus, avoiding its retention in the ER/Golgi membrane, without interfering in the KPNA2 chaperon activity of carrying the STAT1 complex to the nucleus (Fig. 2c). In this regard, CIGB-300 could exhibit an additional effect to that of other CK2 antagonists that target CK2.

### Interfering NUP98 hijacking through the interaction of CIGB-300 with the Orf6 C-terminus

We analyzed the proteomic expression data of Bojkova et al. (2020) and found that B23 exhibits the highest positive correlation with the expression profile of viral proteins (Additional file 2: Fig. S1). The SARS-CoV virus N protein was found to interact with the B23 protein (Zeng et al. 2008). In spite of this, Gordon et al. (2020) did not report a direct interaction of B23 with viral proteins. Searching for indirect interactions, we intersected the set of interactors of B23 with the 322 proteins found by Gordon et al. (2020) that interact with viral proteins. Twenty-one host proteins resulted from this intersection, among which the Nuclear Pore Complex protein 98 (NUP98) was the only one that interacted with Orf6, the viral protein with the highest expression correlation to B23.

Bouhaddou et al. (2020) determined that phosphorylation of NUP98 at Ser888 increased during viral infection. The sequence around Ser888 is D**S**DEEE, which fulfills the phospho-acceptor motif of CK2. Additionally, Franchin et al. (2015) found the phosphorylation of Ser888 to be altered by a CK2 inhibitor (according to data downloaded from the PhosphoSitePlus web site). NUP98 is part of the Nuclear Pore Complex (NPC), responsible for the transport of biomolecules between the nucleus and the cytoplasm. Bouhaddou et al. (2020) suggested that the SARS-CoV-2 infection-induced phosphorylation of NUP98 might prevent the export of mRNAs through the nuclear pore, a similar mechanism to those used by other viruses to increase the transfer of viral RNA in the cytoplasm. The binding of CIGB-300 to the Ser888 phospho-acceptor site of NUP98 could prevent its phosphorylation and restore the translocation of the host mRNA to the cytoplasm.

Moreover, Gordon et al. (2020) found that Met58 and acidic residues Glu55, Glu59 and Asp61 are highly conserved in Orf6 homologs and are part of a putative NUP98/RAE binding motif. Miorin et al. (2020) found that SARS-CoV-2 infection can block the nuclear translocation of STAT1 and STAT2. Orf6 exerts this anti IFN-I activity by hijacking NUP98. Orf6 directly interacts with NUP98 at the NPC through its C-terminal end. A Met58Arg mutant in the Orf6 C-terminal region impairs this interaction and abolishes the IFN-I antagonistic effect (Miorin et al. 2020).

The interactions of Orf6 with KPNA2 and NUP98 have been reported to interfere with IFN signaling (Frieman et al. 2007; Miorin et al. 2020). In both cases the C-terminal domain of Orf6 was responsible for the interaction, in which mutations in this region abolished the anti-IFN activity. The possible binding of CIGB-300 to the CK2 phospho-acceptor site Ser50 in Orf6 could impair the interaction with both KPNA2 and NUP98 and, to a certain extent, restore IFN signaling.

### CIGB-300 downregulates host protein phosphosites that are consistently activated by SARS-CoV-2

We then compared the phosphoproteomic studies of SARS-CoV-2 infection in Vero E6 (Bouhaddou et al. 2020), Caco-2 (Klann et al. 2020), iAT2 (Hekman et al. 2020) and A549 (Stukalov et al. 2021) cell lines with that of Perera et al. (2020) on the kinase antagonistic effect of CIGB-300 in the H125 cell line.

**Fig. 4:**
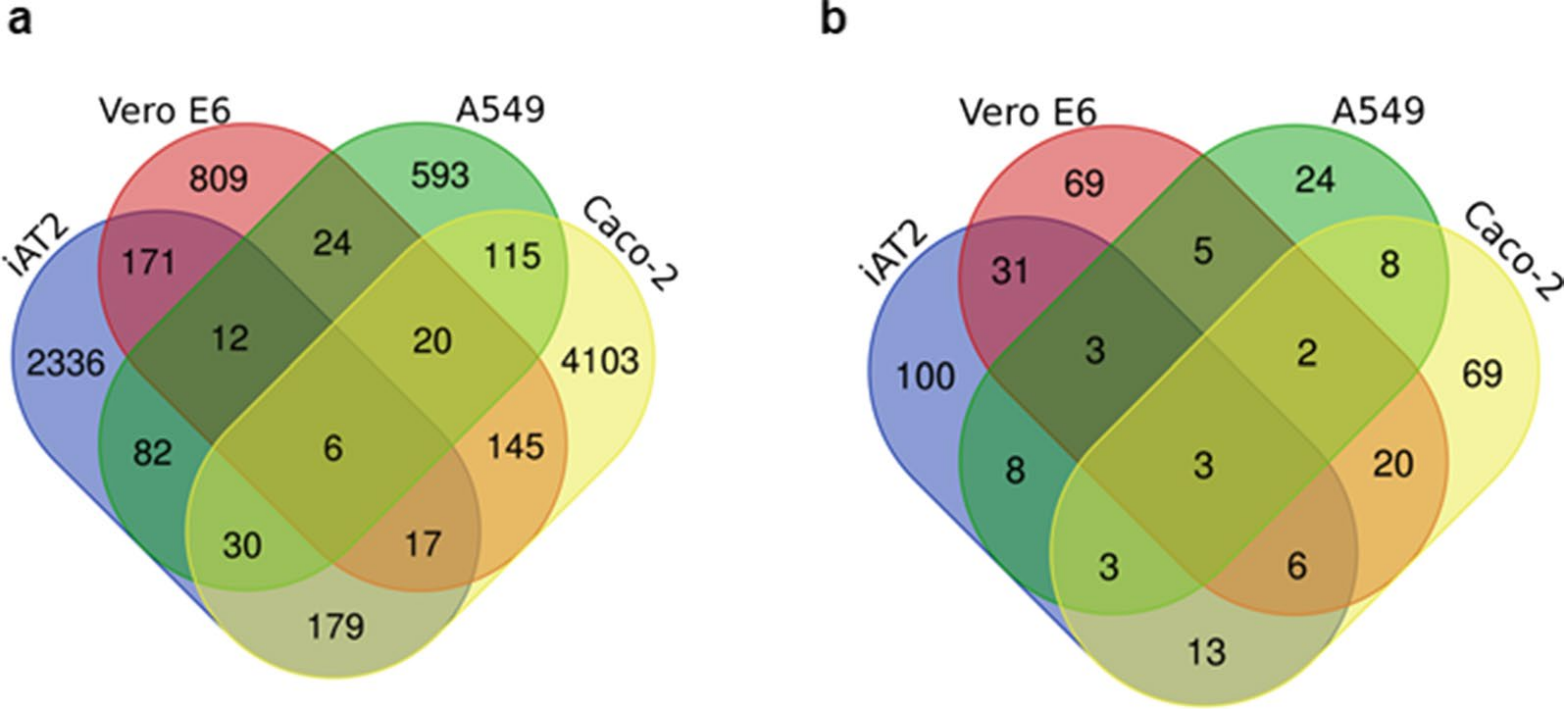
Upregulated phosphorylation sites by SARS-CoV-2 infection and its intersection with the CIGB-300 downregulated phospho-acceptor sites.** a** Venn diagram showing unique phosphorylation sites identified as upregulated in A549, VeroE6, iAT2 and Caco-2 cell lines. **b** Venn diagram showing unique phosphorylation sites identified as upregulated in A549, VeroE6, iAT2 and Caco-2 cells and downregulated by the action of CIGB-300 in the H125 cell line

First, we combined the results of the four studies at the level of the phosphorylation sites and we found 8642 sites that were upregulated in at least one of the studies (Venn diagram in Fig. 4a). As noted by Hekman et al. (2020), few differentially regulated proteins coincide in all four studies. Indeed, we found only six phosphosites that were upregulated by SARS-CoV-2 infection in the four cell lines.

Afterwards, we intersected the data on SARS-CoV-2 infection with that of the phosphorylation sites that were downregulated with the CIGB-300 treatment of the H125 cell line, resulting in 364 sites (see Fig. 4b). Of the six sites that were found to be upregulated in the four phosphoproteomic studies, half of them were downregulated by CIGB-300 (MATR3_S188, SQSTM1_S272 and DIDO1_S1456). These three proteins have in common that they have dozens of phosphorylation sites. Of the three sites, only MATR3_S188 matches the CK2 phospho-acceptor motifs, and is more likely to be directly targeted by CIGB-300. Since CK2 phosphorylates many transcription factors, nuclear proteins implicated in gene transcription and proteins with functions in signaling networks (Pinna 2002), the other two sites may be indirectly inhibited by CK2. Additionally, Perera et al. (2020) reported that only 24% of the sites inhibited by CIGB-300 were known or candidate CK2 substrates and a significant number of them were proteins containing the CK2 consensus in another position, or they were substrates of kinases regulated by CK2. We consider that these three phospho-acceptor sites can be downregulated by CIGB-300 in Covid-19 patients.

MATR3 is a nuclear matrix protein with 36 phosphosites according to UniProt annotations. MATR3 has multiple functions in DNA/RNA processing; it contains two RNA recognition Motifs and two Zinc Finger domains. It was proposed that it could stabilize mRNA species, play a role in the regulation of DNA virus-mediated innate immune response (Salton et al. 2011) and it could be associated to splicing regulation (Yamaguchi and Takanashi 2016). In HIV-infected cells, Sarracino et al. (2018) found that MATR3 was essential for RNA processing. MATR3 phoshorylation was found to greatly enhance its DNA binding ability (Hibino et al. 1998, Malik and Barmada 2021). Its involvement in amyotrophic lateral sclerosis (ALS) is well documented (Johnson et al. 2014). ALS is a disease that causes muscle weakness and respiratory failures, which are also symptoms in the Covid-19 patients. The interference of CIGB-300 on the virus-infection induced phosphorylation of MATR3 may play a role in reducing its effect on the attenuation of the immune response and its involvement in viral RNA processing.

SQSTM1 has several phosphorylation sites, of which Ser272 is the only one that is significantly activated by SARS-CoV-2 in the four phosphoproteomic studies. At positions + 4 and + 5 relative to SQSTM1_S272 there are two putative CK2 phospho-acceptor residues, Ser276 and Ser277. Due to the proximity in the sequence, CIGB-300 can bind to either of them and may interfere with Ser272 phosphorylation. SQSTM1 also has a CK2 phospho-acceptor site at Ser403, which is located in a disordered segment without a solved 3D structure for which reason it was impossible to estimate how distant it was from Ser272, although it could also affect Ser272 phosphorylation. Zhang et al. (2018) found that the phosphorylation of SQSTM1 at Thr269 and Ser272 by MAPK13 promotes the transport of microaggregates to the microtubule organizing center to form aggresomes, which are later degraded through autophagy. Gao et al. (2016) also showed that SQSTM1 phosphorylation increases the ability to sequester ubiquitinated proteins, leading them into aggresomes, thus playing an important role in aggresome formation. Stukalov et al. (2021) revealed a significant reduction of autophagy flux produced by ORF3, which combined with the augmented microaggregates transport due to SQSTM1 phosphorylation, leads to the accumulation of aggresomes.

Several studies have reported the role of SQSTM1 accumulation and aggresome formation in lung related diseases. Tran et al. (2015) demonstrated the role of aggresome formation induced by smoking in chronic obstructive pulmonary disease (COPD). They found a significantly higher accumulation of SQSTM1 in smokers as compared to nonsmokers, and an increased severity of COPD. Wu et al. (2020) found that the accumulation of SQSTM1 plays a critical role in airway inflammation induced by nanoparticles.

Cystic fibrosis (CF), is caused by mutations within the gene encoding the cystic fibrosis transmembrane conductance regulator (CFTR), which results in defective autophagy, causing the accumulation of CFTR containing aggregates (Luciani et al. 2011). SQSTM1 knockdown favored the clearance of defective CFTR aggregates (Luciani et al. 2010).

The inhibition by CIGB-300 of SQSTM1 phosphorylation at Ser272 may reduce the accumulation of aggresomes, and thus attenuates lung inflammation and the fibrosis induced by viral infection.

DIDO1 (death inducer-obliterator 1, also called death-associated transcription factor 1 DATF1) is a protein involved in apoptosis that has also been involved in the progression of several types of cancer (Garcia-Domingo et al. 1999, Lerebours et al. 2021, Li et al. 2020a, Xiao et al. 2020). DIDO1 has 2240 amino acids that possess 92 phosphosites, according to the data we downloaded from PhosphoSitePlus. We found that 16 of these sites match the CK2 phospho-acceptor motif described by Pinna (2002). According to UniProt annotations, two thirds of the protein residues are located in disordered regions. One of these regions extends from residue 1453 to 1472, containing Ser1456. The segment from 1428 to 1497, embracing the disordered regions with the addition of 25 residues at both ends, and including the three putative CK2 phospho-acceptor sites closer to Ser1456, was submitted to the Swiss-Model server. The predicted 3D model consists of two anti-parallel alpha helixes, which are connected by a loop comprising the disordered region (see Additional file 3: Fig. S2). Residues Ser1456 and Ser1471, located at the beginning and at the end of the loop, are however spatially near. The other two putative CK2 sites, Thr1432 and Thr1440, are at the beginning of the N-terminal alpha helix and are more spatially distant from Ser1456. The binding of CIGB-300, a 25-residue peptide, to Ser1471 may interfere in the phosphorylation of Ser1456. The role of DIDO1 in the course of viral infection is not clear, but the induction of apoptosis by viral proteins is documented (Kalantari et al. 2020; Lai et al. 2019, Okamoto et al. 2017), and DIDO1 may be activated by the apoptosis pathway through phosphorylation. CIGB-300 can interfere this activation.

### CIGB-300 at the early stage of SARS-CoV-2 infection

We examined kinase activity from the earliest stages of viral infection by analyzing the phosphoproteomic data of Bouhaddou et al. (2020) at the 2 h and 4 h time points, and that of Hekman et al. (2020), at the 1 h and 3 h time points.

**Fig. 5:**
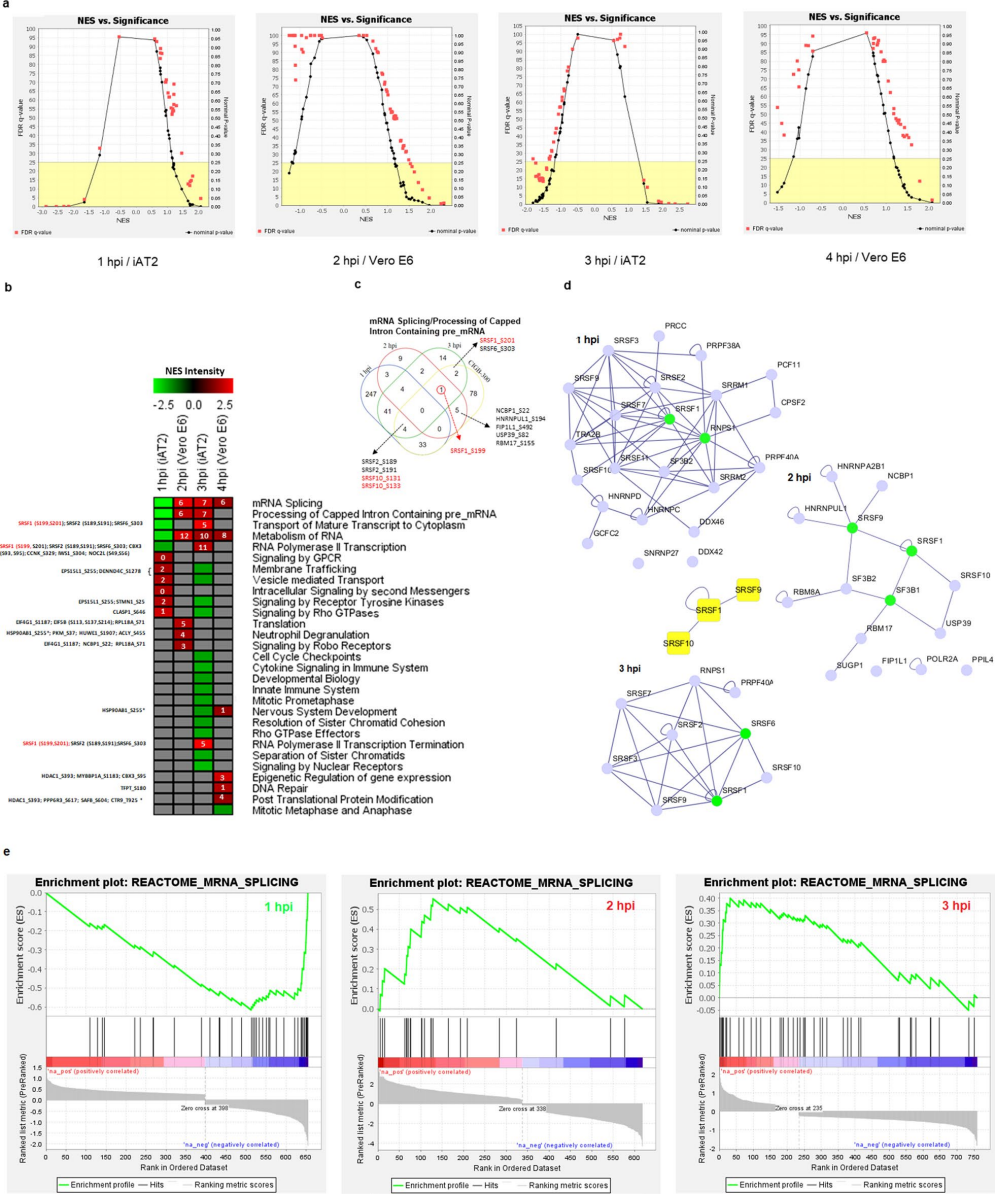
GSEA analysis of the Reactome pathways of proteins with altered phosphorylation at the early stages of infection. **a** NES plotted against the significance of Reactome pathways generated by GSEA at 1 h, 2 h and 3 h after infection. The FDR q-value of a pathway is plotted with a red box, while the corresponding p-value is plotted with a black box. **b** Heat map of the NESs of the Reactome pathways resulting from the GSEA analysis. The four columns corresponds to the 1 h and 3 h time points of the iAT2 cell (Hekman et al. 2020) and the 2 h and 4 h time points of Vero E6 cells (Bouhaddou et al. 2020) after the infection by SARS-CoV-2. The numbers in small rectangles on the heat map indicate the number of proteins in the CES that contain phosphosites upregulated by SARS-CoV-2 infection and inhibited by CIGB-300 (Perera et al. 2020). **c** Venn diagram for sets of phosphorylation sites of proteins in the CES for “mRNA Splicing/Processing of capped intron containing pre-mRNA” Reactome pathways at 1 h, 2 h and 3 h time points or that were inhibited by the action of CIGB-300 according to Perera et al. (2020). The sites upregulated in one of the three time points and inhibited by CIGB-300 are listed. **d** PPI—Networks of proteins in “mRNA Splicing” CES for 1 h, 2 h and 3 h post infection time points. Shown in green are the nodes with the highest degrees; at the center, in yellow, are the nodes representing proteins found in all sub-networks. **e** Profile of the Running ES Score for “mRNA Splicing” gene sets at 1 h, 2 h and 3 h post infection. The middle panel of the plots shows the positions of the members of the gene set on the ranked list. The bottom panel shows the value of the ranking metric (phosphorylation changes) in a descending order

GSEA analysis with protein sets ranked by phosphorylation changes was performed to identify enriched REACTOME pathways. Figure 5a shows the plots of the normalized enrichment scores (NES) vs. FDR q-values and Nominal p-values of Reactome pathways. In these graphs, the black points located at both ends of the x-axis correspond to the p-values of the events of highest or lowest NES, a value that reflects the level of enrichment for each pathway evaluated. Positive and negative NES represent the enrichment at the top and bottom of the pre-ranked list, respectively. The most significant pathways (FDR < 5%) at 1 h are predominantly downregulated (negative NES), while at 2 h and 3 h they are upregulated (positive NES). Figure 5b shows the heat map of statistically significant pathways at each time point. After one hour of infection we observed a clear initial inhibition of the host protein synthesis machinery, reflected in the inactivation of several phosphorylation sites of proteins involved in RNA metabolism events, such as “mRNA Splicing” and the “Pre-processing of capped intron containing mRNA” (Fig. 5b, light-green color in the heat map). This inactivation is reverted by the activation of these same biological events at 2 h and 3 h (Fig. 5b and e). The phosphosites listed are those belonging to proteins from the core enrichment set (CES) of each enriched pathway and those that were identified as having been inactivated by CIGB-300 in Perera et al. (2020). The Venn diagram in Fig. 5c shows the comparison of phosphosylation sites from proteins in the “mRNA Splicing/Processing of capped intron containing pre-mRNA” pathway that were regulated at 1 h, 2 h and 3 h after infection, or inhibited by the action of CIGB-300. Of all those sites, SRSF1_S199 was the only site upregulated at 2 h and 3 h. SRSF1_S201 was upregulated at 3 h as well as some other SRSF protein phosphosites (Fig. 5c). The interaction networks of proteins in these CESs for each time point are shown in Fig. 5d. SRSF proteins are highly connected and predominant in the three networks.

SRSFs are RNA binding protein (RBP) splicing factors that belong to the family of S/R rich proteins. Rogan et al. (2020) proposed a molecular mechanism for viral-RNA pulmonary infections based on protein expression and RBP binding site pattern analysis. They compared the distribution of RBP binding motifs in several viral genomes including SARS-CoV-2, Influenza A, HIV-1 and Dengue. These authors identified strong RBPs binding sites in the SARS-CoV-2 genome. After infection, as the number of SARS-CoV-2 genomes increases, the proportion of the SRSFs bound to the viral genome compared to the host transcriptome also increases. As the virus replicates in the cytoplasm, newly synthetized SRSF1 molecules are bound by the viral RNA and retained there, resulting in the formation of R-loops in the nucleus due to a reduction of the RBP import. Rogan et al. (2020) suggested that this R-loop induced apoptosis could contribute to the spreading of viral particles to neighboring pneumocytes causing a deterioration of lung functions.

Phosphorylation plays an important role in the function of SRSF proteins. SRPK1 kinase was shown to phosphorylate multiple serine residues in the SR rich domain of SRSF1 (Hagopian et al. 2008; Mole et al. 2020), promoting its nuclear import where it plays an important role in RNA stability (Li and Manley 2005) and alternative splicing (Ghosh and Adams 2011). CK2 was found to be the major kinase phosphorylating SRPK1 and this phosphorylation occurs mainly at Ser51 and Ser555, resulting in a sixfold activation of the enzyme (Mylonis and Giannakouros 2003). After SARS-CoV-2 infects AT2 cells, Ser51 is activated at 3 h and 6 h (Hekman et al. 2020).

**Fig. 6:**
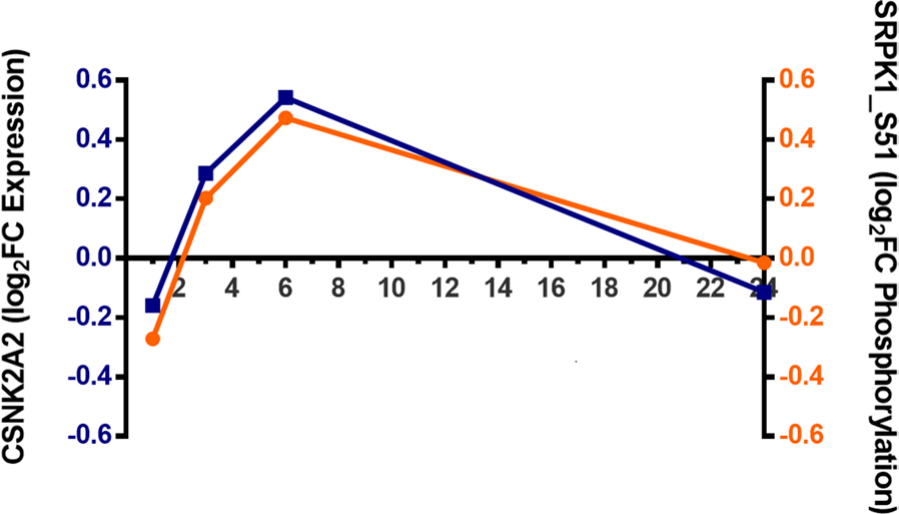
CK2 expression and SRKP1_S51 site phosphorylation profiles

Figure 6 shows the expression profile of CK2 and the levels of phosphorylation of the SRPK1_S51 site, according to data from Hekman et al. (2020). A clear correlation is observed between the amount of CK2 kinase and the phosphorylation activation of this phospho-acceptor site, which is another fact supporting the role of CK2 on the activation of SRPK1 during SARS-CoV-2 infection.

These results are consistent with previous reports predicting an extensive reshaping of splicing pathways by SARS-CoV-2 infection (Bojkova et al. 2020, Klann et al. 2020). SRSF1 is an important element of this splicing machinery that is clearly used by SARS-CoV-2 for its own replication and translation.

The increasing amount of SRSF1 bound to the viral genome as the infection progresses, is a clear indication of its role in viral RNA processing. Phosphorylation is an important mechanism that controls SRSF1 function.

Taking into account all the above mentioned, we suggest that CIGB-300 may interfere in the phosphorylation of SRSF1 by targeting the SRPK1 kinase. It can thus perturb the role of SRSF1 in SARS-CoV-2 protein synthesis.

### Infection-induced protein–protein interactions could be perturbed by CIGB-300

**Fig. 7:**
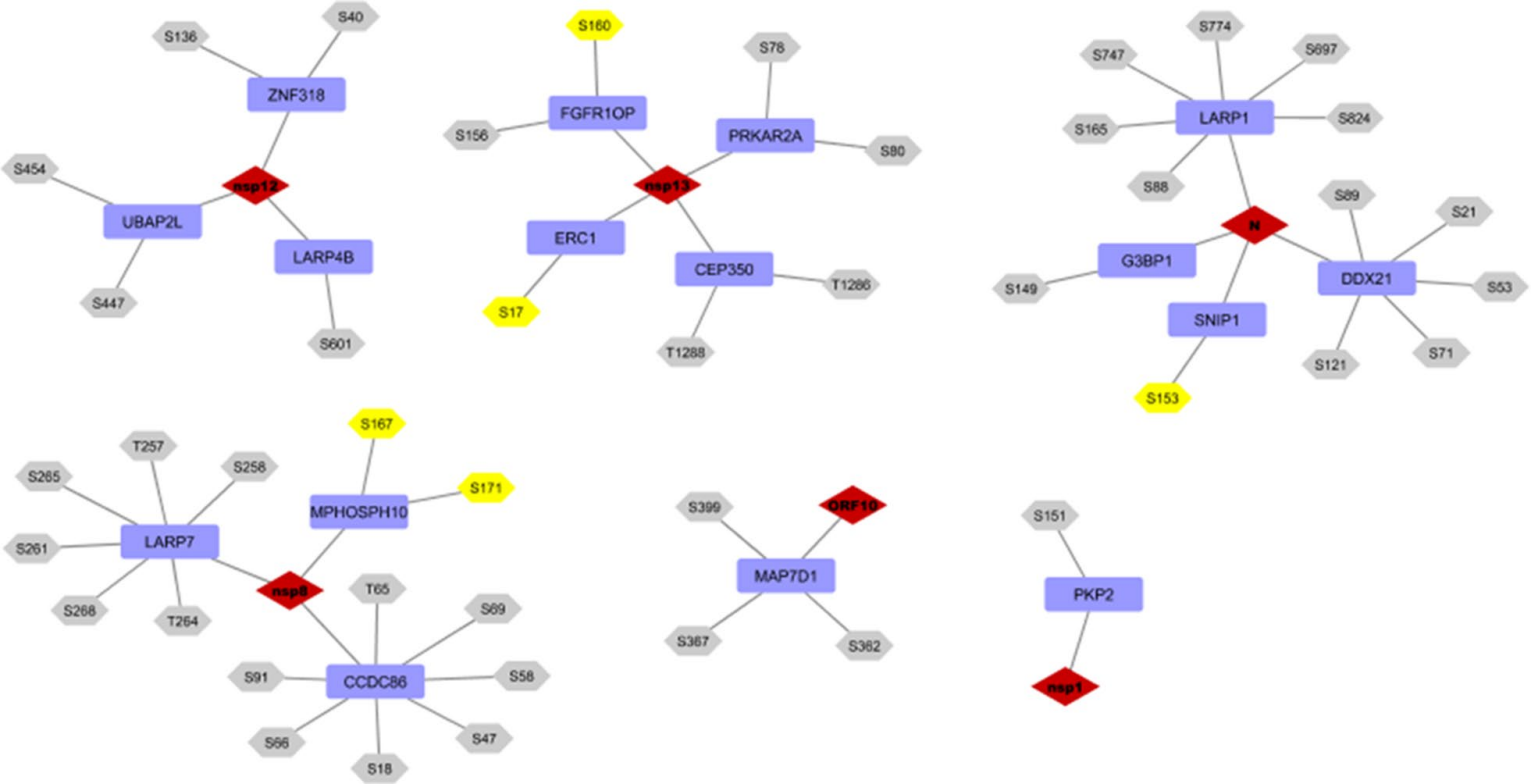
Viral protein interactions with host proteins having phosphorylation sites inhibited by CIGB-300. The rhombus in red represents viral proteins, rectangles in blue represent host proteins, and hexagons represent phospho-acceptor sites. The sites in yellow are those whose phosphorylation was increased by SARS-CoV-2 infection

Next, we compared the host-viral PPI reported by Gordon et al. (2020) with phosphoproteomic data from Perera et al. (2020) on the identification of CK2 substrates that are significantly inhibited by CIGB-300. Figure 7 shows virus-host interactions from Gordon et al. (2020) in which host proteins contain phospho-acceptor sites that were inhibited by the CIGB-300 treatment (highlighted in yellow). In this network, several proteins are related to RNA processing and transcription (LARP1, LARP7, LARP4B), supporting the results already mentioned. The binding of CIGB-300 to the phospho-acceptor sites of host proteins, thus inhibiting their phosphorylation, may perturb the binding by viral proteins and consequently the viral life cycle.

**Fig. 8:**
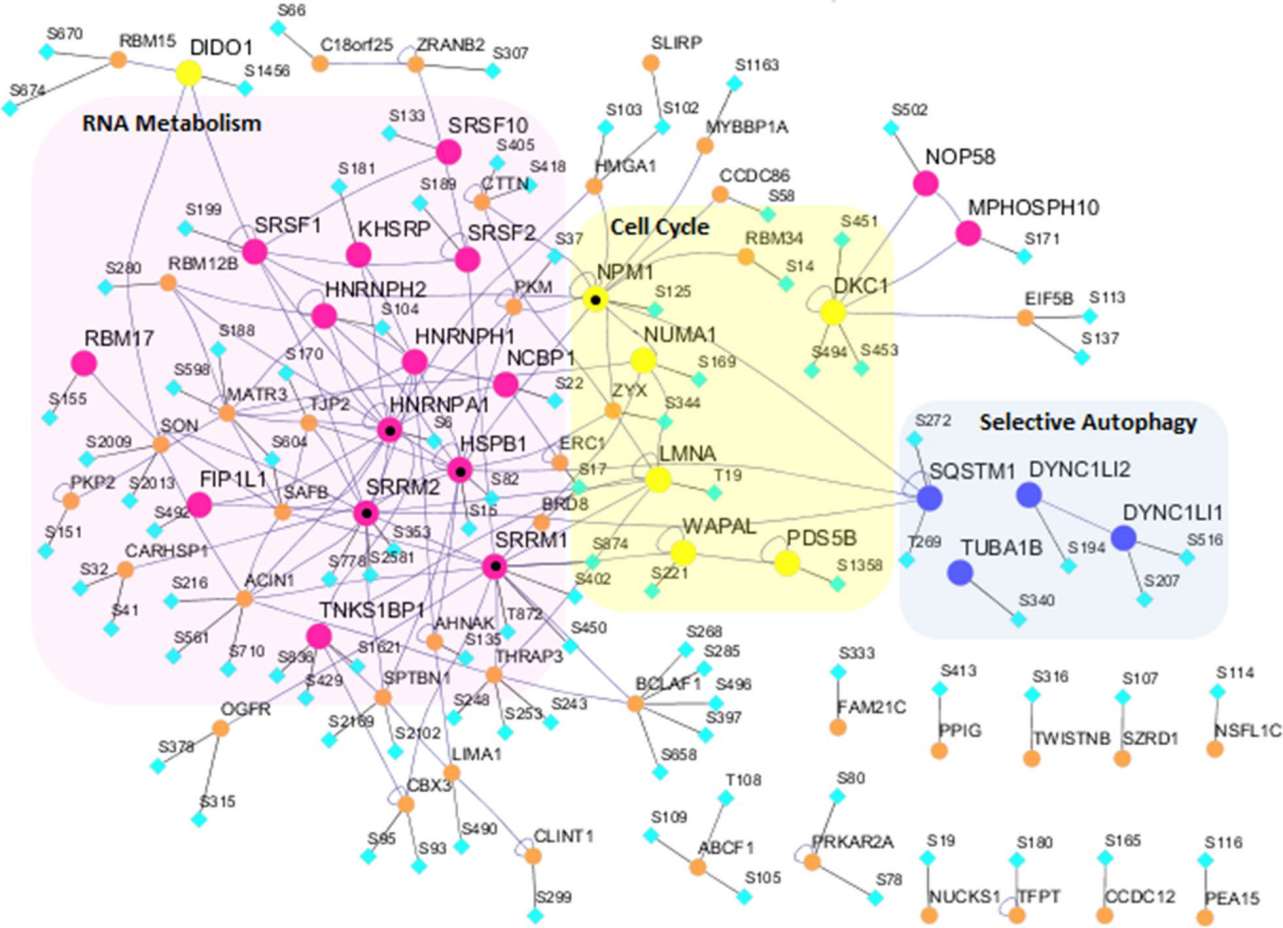
Network of proteins with phosphosites activated in at least two SARS-CoV-2 studies and inhibited by CIGB-300. The phospho-acceptor sites inhibited by CIGB-300 are shown for each protein. Proteins involved in more significant pathways are grouped and colored: mRNA metabolism (pink circle), Cell Cycle (yellow circle) and ‘Selective Autophagy’ (blue circle). The five nodes with the highest degree (HNRNPA1, HSPB1, SRRM2, NPM1 and SRRM1) are labeled with (•)

Evaluating how CIGB-300 may interfere in the host-host protein interactions involved in virus-induced mechanisms, we found that there were 68 proteins (henceforth referred to as SC2_300 set) with activated phospho-acceptor sites in at least two of the four phosphoproteomic studies, which were inhibited by CIGB-300 (see Additional file 4: Table S2). The PPI network built with these proteins is shown in Fig. 8. Most of the nodes in the network are interconnected, indicating potentially functional relations of biological significance among them. Proteins are grouped by mRNA metabolism, Cell Cycle, and Selective Autophagy pathways, identified as significant by a Reactome enrichment analysis (see Additional file 5: Table S3). The five proteins with the highest degree are also highlighted. These include HNRNPA1, HSPB1, SRRM2, and SRRM1, which are involved in mRNA metabolism, thus corroborating the potential impact of CIGB-300 in viral replication and transcription. The fifth protein was B23/NPM1, which was identified as a major target of CIGB-300 in cancer cells, but also, as a relevant target for antiviral therapies (Lobaina and Perera 2019, Nouri et al. 2015, Perera et al. 2009). Out of these five proteins, HSPB1 (alias HSP27) is the only one without a CK2 phospho-acceptor motif, as shown by Borgo et al. (2018), who demonstrated that HSPB1 is not a CK2 substrate. Instead, their results showed that the HSPB1 protein expression is regulated by CK2. They also found that this expression depends on the integrity/activity of the protein kinase CK2 holoenzyme. Perera et al. (2020) reported that CIGB-300 impairs holoenzyme-dependent phosphorylation by directly binding to CK2α. When analyzing the data from a proteomic study on SARS-CoV-2 infection (Bojkova et al. 2020) we detected a peak of HSPB1 abundance at 6 h after infection, while in the data from a phospho-proteomic study (Hekman et al. 2020) we found a peak of the CK2 enzymatic activity at that same time point. This is in line with the findings of Borgo et al. (2018). In accordance with the above, the CIGB-300 treatment may produce the downregulation of HSPB1 by impairing CK2 holoenzyme activity.

As shown in the network (Fig. 8), the HSPB1 heat shock protein is one of those high degree nodes. HSPB1 was found to be overexpressed in idiopathic pulmonary fibrosis (IPF) patients. It activates pro-fibrotic mechanisms and was therefore suggested as a target to treat IPF (Park et al. 2016; Wettstein et al. 2013).

### Human phenotypes involving kinase activity induced by SARS-CoV-2 that are potentially targeted by CIGB-300

**Fig. 9:**
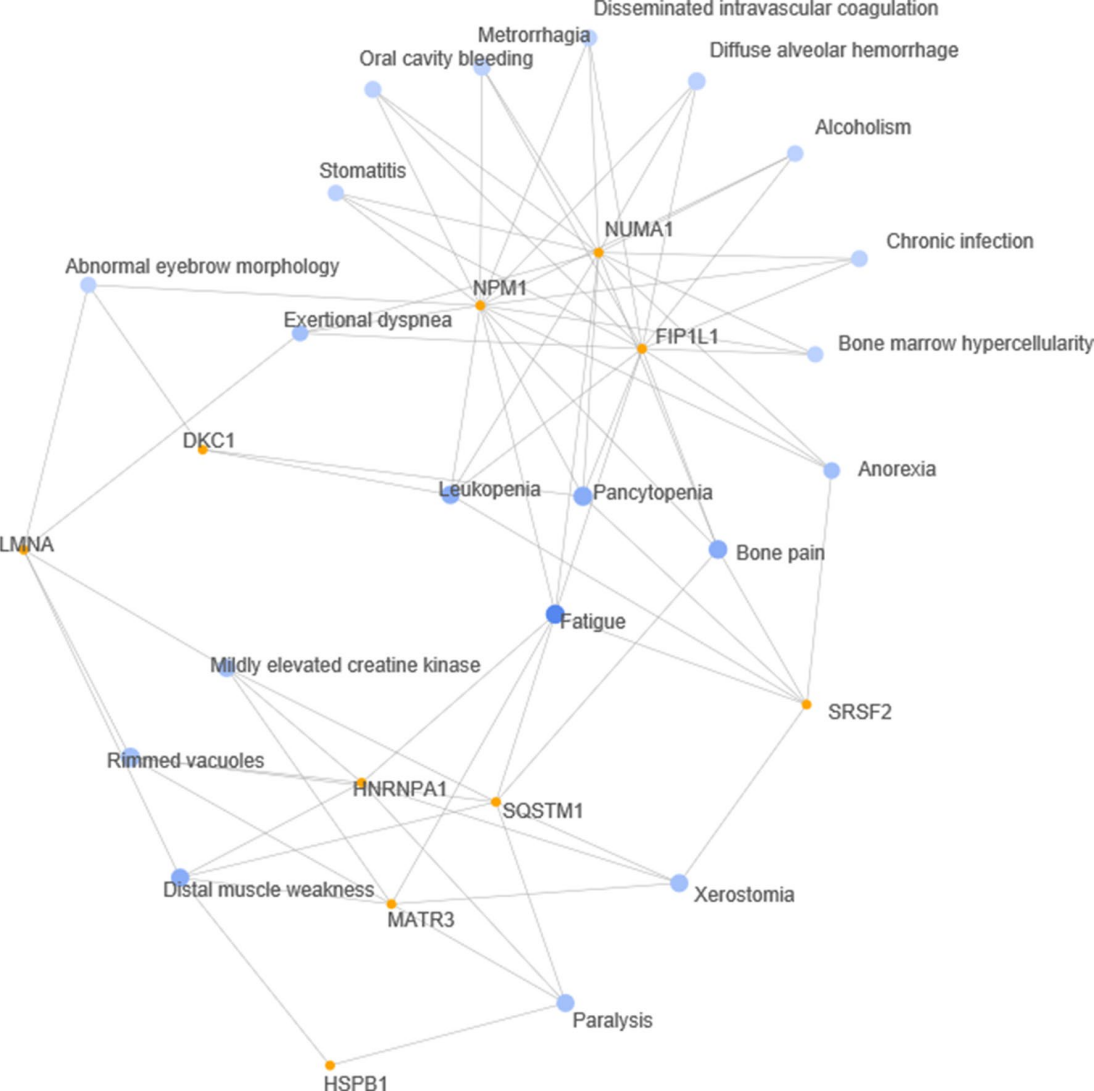
Network of enriched phenotypes in proteins with phosphosites activated in at least two SARS-CoV-2 studies and inhibited by CIGB-300. Genecodis functional annotation tool was used for the enrichment analysis and to generate the network of the top 20 enriched phenotypes. Nodes in blue represent enriched phenotypes and nodes in orange represent the proteins associated to these phenotypes

We built a network with the top 20 most enriched human phenotypes in the SC2_300 set, using the GeneCodis tool (see Fig. 9 and Additional file 6: Table S4). The network can be divided into two main sub-networks, one related to muscular disorder phenotypes that include paralysis, distal muscle weakness, rimmed vacuoles, mildly elevated creatine kinase and fatigue. The second sub-network groups the phenotypes related to respiratory (exertional dyspnea, diffuse alveolar hemorrhage), bleeding (metrorrhagia, oral cavity bleeding) and coagulation (disseminated intravascular coagulation) disorders.

The phenotypes in the first sub-network are all associated to HNRNPA1, MATR3 and SQSTM1 genes, and were also reported as Covid-19 symptoms (Akbar et al. 2021, Chan et al. 2020, De Giorgio et al. 2020, Townsend et al. 2020, Versace et al. 2021). For example, high creatine kinase levels are associated to the prediction of a poor outcome (Akbar et al. 2021) and persistent fatigue is a common symptom in Covid-19 patients (Townsend et al. 2020). As mentioned above, MATR3 and SQSTM1 have phosphosites that were activated in all four phosphoproteomic studies we analyzed, and HNRNPA1 was the node showing the highest degree in the built PPI network. Rimmed vacuoles, the most significant of the enriched phenotypes, are found in areas of destruction of muscle fibers. The fatigue phenotype is located somewhere in the interface between the two sub-networks and is connected to the three genes mentioned above, and also to the three genes that are in the core of the second sub-network: B23/NPM1, FIP1L1 and NUMA1. All of the phenotypes in the second sub-network have been identified in Covid-19 patients (Levi and Iba 2021, Singh and Schwartz 2020).

## Discussion

Despite the progress shown by the development and extensive use of vaccines, there is still no definitively effective therapeutic treatment against SARS-CoV-2. The emergence of increasingly transmissible and aggressive mutated variants of the virus, demands continuous efforts in the search for novel therapies to reduce the risk of evolving toward severe stages and the death of patients.

Targeting the mechanisms of the host cells, commonly hijacked by the viruses for reproduction and spreading, while consequently damaging host cell functions, is a recognized strategy to confront present and future challenges of viral epidemics.

Phosphorylation is one of the mechanisms that is highly altered in human cells immediately after the entry of the virus, contributing to the hijacking of multiple cellular processes.

We evidenced the potential incidence of CIGB-300 in SARS-CoV-2 induced N protein phosphorylation and localization that would alter N protein binding properties and its essential role in viral capsid assembly.

Coronavirus nucleocapsid N proteins play an essential role in the virus cell cycle. Its dimerization and binding to the viral genomic RNA is the first step for virion particle assembly. N protein is also involved in viral genomic RNA synthesis (Wu et al. 2014) and was also identified as an antagonist of type I interferon signaling (Li et al. 2020b).

One distinguishing pattern of SARS-CoV-2 infection is a decreased transcriptional response of type I/III interferon-induced genes. These genes are relevant for the antiviral host response to virus infection. Orf6 is one of the viral proteins identified as having an antagonistic effect on this response, in particular its C-terminal end. Our results support the likely binding of CIGB-300 to a CK2 phospho-acceptor site at residue Ser50 near its C-terminal end, which may help restore IFN signaling.

SARS-CoV-2 infection is also characterized by the induction of cytokines that lead to a strong inflammatory response. The accumulation of SQSTM1, due to an increase in its phosphorylation levels, plays an important role in airway inflammation and fibrosis. The binding of CIGB-300 to the Ser272 of SQSTM1, may reduce aggresome accumulation and consequently reduce lung damage in Covid-19 patients.

One of the mechanisms that viruses use to increase their replication and translation is the hijacking of the host proteins involved in RNA metabolism. As in other reports, our analysis of multiple phosphoproteomic studies also found these same phenomena in SARS-CoV-2 infected cells starting at the early stages of the infection. A significant increase of kinase activity is observed, which involves CK2 phospho-acceptor sites in proteins implicated in “mRNA Splicing” and the “Processing of capped intron containing pre-mRNA”. At the same time, we identified that several of these phospho-acceptor sites are inhibited by the action of CIGB-300. In particular, several members of the SRSF family of proteins, which are essential splicing factors, are targeted by CIGB-300. This supports the potential role of CIGB-300 in perturbing the hijacking of host proteins at early stages of viral infection.

Our study revealed how the use of CIGB-300 may participate in attenuating some of the phenotypes frequently observed in Covid-19 patients, particularly those involved in muscle, bleeding, coagulation and respiratory disorders.

We observed other remarkable findings, once again highlighting B23 as a relevant player in a viral infection, now in the context of SARS-CoV-2. Firstly, it was the host protein with the highest correlation of expression to viral proteins, in particular to Orf6. Secondly, we identified B23 as a highly connected node in a network of proteins that are consistently upregulated by SARS-CoV-2 infection and inhibited by CIGB-300, which is related to the Cell Cycle pathway (Fig. 8). Thirdly, it was part of a phenotype network related to respiratory, bleeding and coagulation disorders, which are widely reported symptoms in Covid-19. Previously, Kondo et al. (1997) showed that B23 inhibited DNA-binding and the transcriptional activity of the interferon regulatory factor 1 (IRF1), while Abe et al. (2018) found that B23 regulates the expression of IFN-γ-inducible genes and binds to transcription factors STAT1 and IRF1. The aforementioned indicate that both Orf6 and B23 may play a role in the inhibitory effect of IFN signaling.

On the other hand, it is known that post-translational modifications, such as phosphorylation, are involved in the regulation of molecular chaperone activities (Jovcevski et al. 2015). In particular CK2 phosphorylation was found to play an important role in the B23 chaperon activity (Szebeni et al. 2003).

CIGB-300 may interfere in the B23 chaperon activity by inhibiting phosphorylation and perturbing its interactions with host and viral proteins. For instance, since Orf6 is located in the ER/Golgi membrane and the NPC associated to KPNA2 and NUP98, would it be possible for the B23 chaperone activity to play a role in SARS-CoV-2 infected cells by transporting Orf6 to the ER/Golgi membrane and to the NPC?

It is noteworthy that the CIGB-300 peptide interacted with the B23 protein in MDKB cells infected with a Bovine Coronavirus strain (BCoV) (Ramon et al. 2021). In these cells, several host proteins participating in protein folding, are also involved in the interactomic profile of the CIGB-300 peptide. However, genetic experiments that involve the “gain- and/or lost-of-functions” are required to define a particular role of B23/NPM1 in the context of the ongoing coronavirus infection.

In summary, we support different hypotheses that must be verified in suitable pre-clinical models (see Table 2). Notably, while our findings suggest a clear impact of CK2 inhibitors in viral replication and hijacking strategies, differences may be inferred based on their particular inhibitory mechanisms. For instance, the use of CIGB-300 to impair CK2-mediated signaling in cancer does not mirror the CX-4945 effects in pre-clinical and clinical settings; this could also be expected in viral infections such as those produced by coronaviruses. The fact that CIGB-300 targets both the CK2 enzyme and a subset of its substrates, may imply particular inhibitory effects of protein–protein interactions, as well as in the interference with other nearby post-translational modification sites (Perera et al. 2020), thus resulting in different molecular, cellular and organismal outcomes.

**Table 2:** Summary of main findings

Analysis type	Subject	Identity	Working hypothesis	Experimental clues	References
Individual CK2 sites	Viral proteins	N	CK2 phosphosite inhibition/blockage by CIGB-300 impairs viral replication	Interaction, co-localization, N mRNA and protein expression inhibition by CIGB-300 in a subrogate model	Ramon et al. (2021)
Individual CK2 sites	Viral proteins	Orf6	CK2 phosphosite inhibition/blockage by CIGB-300 restores IFN signaling	Additive/Synergistic profile of CIGB-300 plus IFN alpha	Unpublished
Co-Expression and network propagation	Host and Viral Proteins	B23, Orf6, NUP98	CK2 phosphosite inhibition/blockage by CIGB-300 restores IFN signaling	Additive/Synergistic profile of CIGB-300 plus IFN alpha	Unpublished
Phosphoproteome overlap	Host Proteins	MATR3, SQSTM1, DIDO1	CIGB-300 Multitarget effect impairing viral transcription/splicing, inflammation, immunoresponse and apoptosis	Pulmonary lesions resolution in CT Phase I	Cruz et al. (2020)
Enrichment analysis at early viral infection and Kinase activity profiles	Host proteins	SRPK1 (SRSF1 and SRSF-2,6,10)	CIGB-300 impairs SRSF1 role in viral protein synthesis	None, to be evaluated in preclinical settings	NA
PPIs vs H125 Phosphoproteome	Host and Viral Proteins	Several proteins (see text and Fig. 7) [LARP1, LARP7, LARP4B]	CIGB-300 impairs Viral RNA processing and transcription	None, to be evaluated in preclinical settings	NA
SARS-CoV-2 vs H125 Phosphoproteome overlap, Network and Enrichment analysis	Host proteins	Several proteins (see text and Fig. 8) [HNRNPA1, HSPB1, SRRM2, SRRM1, B23]	CIGB-300 Multitarget effect impairing on mRNA metabolism, cell cycle and Autophagy pathways	Pulmonary lesions resolution in CT Phase I	Cruz et al. (2020)
Enrichment of Human Phenotypes	Host proteins	HNPRNPA1, MATR3, SQSTM1, B23, FIP1L1, NUMA1	CIGB-300 may relief Covid-19 clinical symptoms	To be evaluated in CT Phase II	NA

## Conclusions

The current study predicts the interference of CIGB-300 in several SARS-CoV-2 infection-induced phosphorylation events that play a role in virus reproduction and spreading. CIGB-300 targets phospho-acceptor sites that are consistently upregulated by SARS-CoV-2 infection. Additional studies are needed to corroborate some of the proposed mechanisms underlying the effect of CIGB-300 in SARS-CoV-2 infection.

## Supplementary Information

**Additional file 1: Table S1.** N protein phospho-acceptor sites reported in four phosphoproteomic studies*.

**Additional file 2: Fig S1.** Bidimensional clustering of most differentially expressed proteins in SARS-CoV-2 proteomic study.

**Additional file 3: Fig S2.** DIDO1 segment 3D model.

**Additional file 4: Table S2.** List of 102 phosphosites activated in at least two of the four phosphoproteomic studies.

**Additional file 5: Table S3.** REACTOME pathway enrichment results.

**Additional file 6: Table S4.** Human phenotype enrichment results.

## Data Availability

All relevant data generated by authors are within the manuscript and its additional information files.

## Abbreviations

GSEA: Gene set enrichment analysis
FDR: False discovery rate
NES: Normalized enrichment score
CES: Core enrichment set
PPI: Protein–protein interactions
NPM1/B23: Nucleophosmin
SRSF1: Serine and arginine rich splicing factor 1
SRPK1: SRSF protein kinase 1
SQSTM1: Sequestosome 1
HSPB1: Heat shock protein family B (small) member 1
NF-κB: Nuclear factor-kappa B
HNRNPA1: Heterogeneous nuclear ribonucleoprotein A1
MATR3: Matrin 3
DIDO1: Death inducer-obliterator 1
KPNA1: Karyopherin subunit alpha 1
KPNB1: Karyopherin subunit beta 1
