# Supplementary material for: Targeting CK2 mediated signaling to impair/tackle SARS-CoV-2 infection: a computational biology approach
Source: Mol Med. 2021 Dec 20;27:161. doi: 10.1186/s10020-021-00424-x (PMC8686809; doi:10.1186/s10020-021-00424-x)
Supplement: Supplementary file 1 — Additional file 1: Table S1. N protein phospho-acceptor sites reported in four phosphoproteomic studies*. [file 10020_2021_424_MOESM1_ESM.pdf]

Supplementary Information:

Table S1. N protein phospho-acceptor sites reported in four phosphoproteomic studies*.

	Phosphosites	Davidson	Bouhaddou	Klann	Hekman
1	S2	X			
2	S23	X	X	X	X
3	T24	X	X		
4	S26		X	X	X
5	S33			X	
6	T76	X	X		
7	S78	X		X	
8	S79	X	X	X	X
9	S105	X	X		
10	Y109			X	
11	T141	X		X	
12	T166	X			
13	S176	X	X	X	
14	S180	X	X	X	
15	S183	X	X	X	
16	S184	X	X	X	
17	S186			X	
18	S187			X	
19	S188			X	
20	S190			X	
21	S194	X	X		X
22	S197		X		X
23	T198	X	X	X	X
24	S201	X	X	X	
25	S202	X	X	X	
26	T205	X	X	X	X
27	S206	X	X	X	X
28	T391	X			
29	S410				X
30	S412				X
31	S413			X	
32	S416				X
33	T417			X	
	Total	20	17	22	11

* the four phosphoproteomic studies are Davidson et al. (2020); Bouhaddou et al. (2020); Klann et al. (2020) and Hekman et al. (2020).
