# Supplementary material for: Targeting CK2 mediated signaling to impair/tackle SARS-CoV-2 infection: a computational biology approach
Source: Mol Med. 2021 Dec 20;27:161. doi: 10.1186/s10020-021-00424-x (PMC8686809; doi:10.1186/s10020-021-00424-x)
Supplement: Supplementary file 2 — Additional file 2: Fig S1. Bidimensional clustering of most differentially expressed proteins in SARS-CoV-2 proteomic study. [file 10020_2021_424_MOESM2_ESM.pdf]

Supplementary Information:

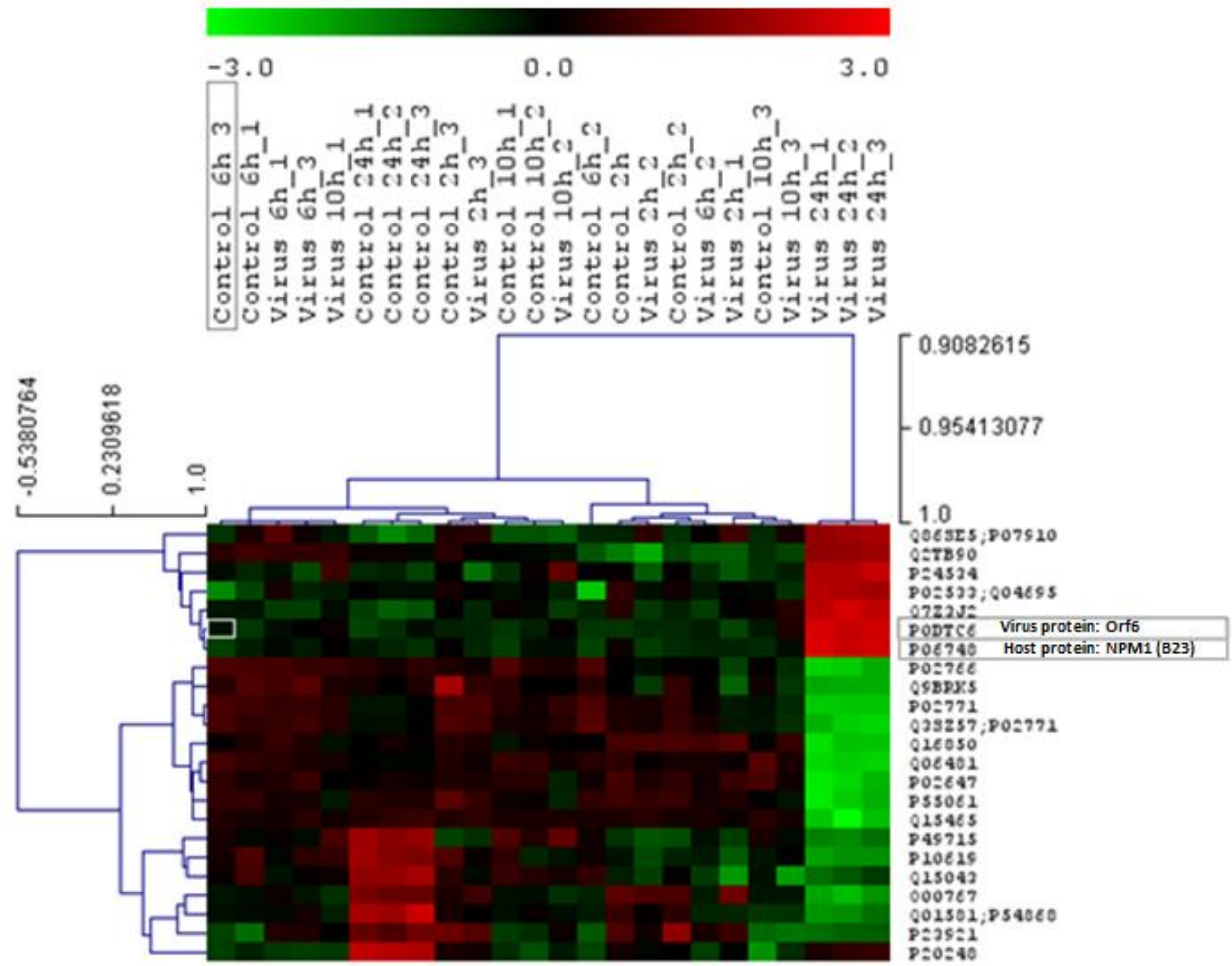

Fig. S1: **Bidimensional clustering of most differentially expressed proteins in SARS-CoV-2 proteomic study.** Horizontal axis represents samples at different time points and vertical axis represent differentially expressed proteins. Rows corresponding to viral Orf6 and host B23 proteins are labeled. Proteomic data: Bojkova et al. (2020).
