# Supplementary material for: Targeting CK2 mediated signaling to impair/tackle SARS-CoV-2 infection: a computational biology approach
Source: Mol Med. 2021 Dec 20;27:161. doi: 10.1186/s10020-021-00424-x (PMC8686809; doi:10.1186/s10020-021-00424-x)
Supplement: Supplementary file 3 — Additional file 3: Fig S2. DIDO1 segment 3D model. [file 10020_2021_424_MOESM3_ESM.pdf]

Supplementary Information:

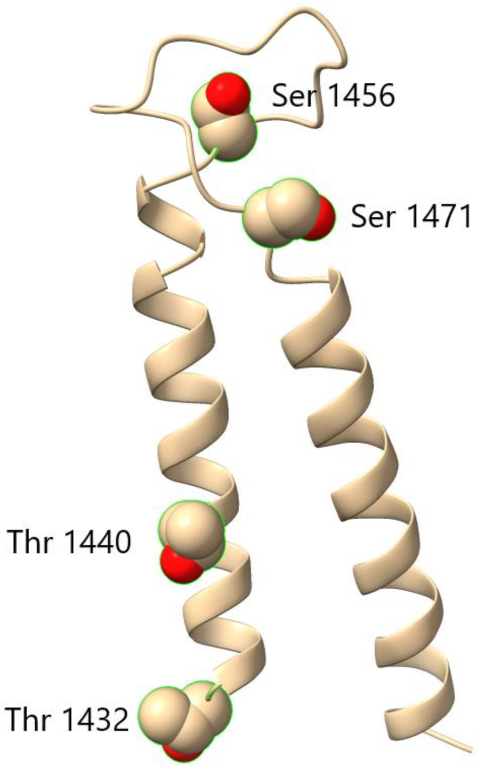

Fig. S2: DIDO1 segment 3D model. 3D model of segment 1428 to 1497 from DIDO1 generated by Swiss-Model server is shown as a ribbon diagram. Thr1432, Thr1440, Ser1456 and Ser1471 are labeled and their atoms shown as spheres.
