# Supplementary material for: Targeting CK2 mediated signaling to impair/tackle SARS-CoV-2 infection: a computational biology approach
Source: Mol Med. 2021 Dec 20;27:161. doi: 10.1186/s10020-021-00424-x (PMC8686809; doi:10.1186/s10020-021-00424-x)
Supplement: Supplementary file 4 — Additional file 4: Table S2. List of 102 phosphosites activated in at least two of the four phosphoproteomic studies. [file 10020_2021_424_MOESM4_ESM.pdf]

Supplementary Information:

Table S2: List of 102 phosphosites activated in at least two of the four SARS-CoV-2 phosphoproteomic studies. Level 1 (in 4 experiments), level 2 (in 3 experiments) and level 3 (in 2 experiments).

		**Phosphosites**		
	**Gene Name**	level 1	level 2	level 3
1	MATR3	S188	S604	S598
2	SQSTM1	S272	T269	
3	DIDO1	S1456		
4	DKC1		S451	S494,S453
5	HSPB1		S82	S15
6	CTTN		S418	S405
7	EIF5B		S113	S137
8	SON		S2009	S2013
9	KHSRP		S181	
10	NUCKS1		S19	
11	RBM15		S670	
12	RBM34		S14	
13	SAFB		S604	
14	SRSF10		S133	
15	TWISTNB		S316	
16	BCLAF1			S285,S397,S496,S268,S658
17	SRRM1			S874,T872,S402,S450
18	SRRM2			S778,S353,S2581
19	DYNC1LI1			S516,S207,S194
20	ABCF1			T108,S105,S109
21	ACIN1			S216,S710,S561
22	TNKS1BP1			S1621,S429,S836
23	THRAP3			S248,S253,S243
24	CARHSP1			S32,S41
25	CBX3			S93,S95
26	HMGA1			S102,S103
27	PRKAR2A			S78,S80
28	SPTBN1			S2169,S2102
29	OGFR			S315,S378
30	AHNAK			S135
31	BRD8			S17
32	C18orf25			S66
33	CCDC12			S165
34	CCDC86			S58

35	CLINT1			S299
36	ERC1			S17
37	FAM21C			S333
38	FIP1L1			S492
39	HNRNPA1			S6
40	HNRNPH1			S104
41	HNRNPH2			S104
42	LIMA1			S490
43	LMNA			T19
44	MPHOSPH10			S171
45	MYBBP1A			S1163
46	NCBP1			S22
47	NOP58			S502
48	NPM1			S125
49	NSFL1C			S114
50	NUMA1			S169
51	PDS5B			S1358
52	PEA15			S116
53	PKM			S37
54	PKP2			S151
55	PPIG			S413
56	RBM12B			S280
57	RBM15			S674
58	RBM17			S155
59	SLIRP			S102
60	SRSF1			S199
61	SRSF2			S189
62	SZRD1			S107
63	TFPT			S180
64	TJP2			S170
65	TUBA1B			S340
66	WAPAL			S221
67	ZRANB2			S307
68	ZYX			S344
