# Supplementary material for: Targeting CK2 mediated signaling to impair/tackle SARS-CoV-2 infection: a computational biology approach
Source: Mol Med. 2021 Dec 20;27:161. doi: 10.1186/s10020-021-00424-x (PMC8686809; doi:10.1186/s10020-021-00424-x)
Supplement: Supplementary file 5 — Additional file 5: Table S3. REACTOME pathway enrichment results. [file 10020_2021_424_MOESM5_ESM.pdf]

Supplementary Information:

Table S3: REACTOME pathway enrichment results.

Pathway identifier	Pathway name	#Entities found	Entities pValue	Entities FDR	Submitted entities found
R-HSA-72172	mRNA Splicing	11	5.99E-09	1.00E-06	SRRM2;RBM17;FIP1L1;NCBP1;HNRNPH1;SRSF1;SRSF2;HNRNPH2;HNRNPA1;SRSF10;SRRM1
R-HSA-72163	mRNA Splicing - Major Pathway	11	3.32E-09	1.00E-06	SRRM2;RBM17;FIP1L1;NCBP1;HNRNPH1;SRSF1;SRSF2;HNRNPH2;HNRNPA1;SRSF10;SRRM1
R-HSA-72203	Processing of Capped Intron-Containing Pre-mRNA	11	8.86E-08	9.83E-06	SRRM2;RBM17;FIP1L1;NCBP1;HNRNPH1;SRSF1;SRSF2;HNRNPH2;HNRNPA1;SRSF10;SRRM1
R-HSA-8953854	Metabolism of RNA	17	3.93E-07	3.26E-05	SRRM2;RBM17;NOP58;NCBP1;SRSF1;HSPB1;SRRM1;FIP1L1;HNRNPH1;DKC1;KHSRP;SRSF2;TNKS1BP1;MPHOSPH10;HNRNPH2;HNRNPA1;SRSF10
R-HSA-72187	mRNA 3'-end processing	5	1.39E-05	0.000931	FIP1L1;NCBP1;SRSF1;SRSF2;SRRM1
R-HSA-73856	RNA Polymerase II Transcription Termination	5	2.76E-05	0.001517	FIP1L1;NCBP1;SRSF1;SRSF2;SRRM1
R-HSA-72202	Transport of Mature Transcript to Cytoplasm	5	0.00011	0.005171	FIP1L1;NCBP1;SRSF1;SRSF2;SRRM1
R-HSA-2555396	Mitotic Metaphase and Anaphase	7	0.000319	0.011829	DYNC1LI1;TUBA1B;DYNC1LI2;LMNA;WAPAL;PDS5B
R-HSA-68882	Mitotic Anaphase	7	0.000312	0.011829	DYNC1LI1;TUBA1B;DYNC1LI2;LMNA;WAPAL;PDS5B
R-HSA-6803529	FGFR2 alternative splicing	3	0.000436	0.014397	NCBP1;HNRNPH1;HNRNPA1
R-HSA-2500257	Resolution of Sister Chromatid Cohesion	5	0.000675	0.020264	DYNC1LI1;TUBA1B;DYNC1LI2;WAPAL;PDS5B
R-HSA-159236	Transport of Mature mRNA derived from an Intron-Containing Transcript	4	0.000858	0.021455	NCBP1;SRSF1;SRSF2;SRRM1
R-HSA-68877	Mitotic Prometaphase	6	0.000804	0.021455	DYNC1LI1;TUBA1B;DYNC1LI2;NUMA1;WAPAL;PDS5B
R-HSA-111465	Apoptotic cleavage of cellular proteins	3	0.001051	0.024172	LMNA;ACIN1;TJP2
R-HSA-68886	M Phase	8	0.001387	0.024969	DYNC1LI1;TUBA1B;DYNC1LI2;NUMA1;LMNA;WAPAL;PDS5B
R-HSA-1640170	Cell Cycle	11	0.001344	0.024969	DIDO1;DYNC1LI1;TUBA1B;DYNC1LI2;NPM1;NUMA1;DKC1;LMNA;WAPAL;PDS5B
R-HSA-2470946	Cohesin Loading onto Chromatin	2	0.001272	0.024969	WAPAL;PDS5B
R-HSA-9663891	Selective autophagy	4	0.001212	0.024969	DYNC1LI1;TUBA1B;DYNC1LI2;SQSTM1
R-HSA-9646399	Aggrephagy	3	0.001923	0.032702	DYNC1LI1;TUBA1B;DYNC1LI2
R-HSA-2468052	Establishment of Sister Chromatid Cohesion	2	0.002129	0.034067	WAPAL;PDS5B
R-HSA-6807878	COPI-mediated anterograde transport	4	0.002363	0.035447	DYNC1LI1;TUBA1B;DYNC1LI2;SPTBN1
R-HSA-75153	Apoptotic execution phase	3	0.002843	0.036963	LMNA;ACIN1;TJP2
R-HSA-68884	Mitotic Telophase/Cytokinesis	2	0.002815	0.036963	WAPAL;PDS5B
R-HSA-72165	mRNA Splicing - Minor Pathway	3	0.003147	0.040922	NCBP1;SRSF1;SRSF2
R-HSA-2467813	Separation of Sister Chromatids	5	0.003459	0.041513	DYNC1LI1;TUBA1B;DYNC1LI2;WAPAL;PDS5B
R-HSA-77595	Processing of Intronless Pre-mRNAs	2	0.004458	0.049040	FIP1L1;NCBP1
R-HSA-6811436	COPI-independent Golgi-to-ER retrograde traffic	3	0.004368	0.049040	DYNC1LI1;TUBA1B;DYNC1LI2
