# Supplementary material for: Targeting CK2 mediated signaling to impair/tackle SARS-CoV-2 infection: a computational biology approach
Source: Mol Med. 2021 Dec 20;27:161. doi: 10.1186/s10020-021-00424-x (PMC8686809; doi:10.1186/s10020-021-00424-x)
Supplement: Supplementary file 6 — Additional file 6: Table S4. Human phenotype enrichment results. [file 10020_2021_424_MOESM6_ESM.pdf]

Supplementary Information:

Table S4: Human phenotype enrichment results.

	Description	Annotation_id	pval	pval_adj	Genes
1	Rimmed vacuoles	HP:0003805	3.62E-08	9.70E-06	MATR3,SQSTM1,LMNA,HNRNPA1
2	Pancytopenia	HP:0001876	6.05E-08	1.22E-05	SRSF2,NUMA1,NPM1,FIP1L1,DKC1
3	Fatigue	HP:0012378	3.18E-08	1.28E-05	MATR3,SQSTM1,SRSF2,NUMA1,NPM1,HNRNPA1,FIP1L1
4	Leukopenia	HP:0001882	1.24E-07	2.00E-05	SRSF2,NUMA1,NPM1,FIP1L1,DKC1
5	Bone pain	HP:0002653	2.60E-08	2.09E-05	SQSTM1,SRSF2,NUMA1,NPM1,FIP1L1
6	Distal muscle weakness	HP:0002460	3.89E-07	3.91E-05	MATR3,SQSTM1,LMNA,HSPB1,HNRNPA1
7	Paralysis	HP:0003470	2.99E-07	4.01E-05	MATR3,SQSTM1,HSPB1,HNRNPA1
8	Diffuse alveolar hemorrhage	HP:0025420	4.62E-07	4.13E-05	NUMA1,NPM1,FIP1L1
9	Xerostomia	HP:0000217	3.80E-07	4.36E-05	MATR3,SQSTM1,SRSF2,HNRNPA1
10	Metrorrhagia	HP:0100608	7.10E-07	5.71E-05	NUMA1,NPM1,FIP1L1
11	Mildly elevated creatine kinase	HP:0008180	8.16E-07	5.96E-05	MATR3,SQSTM1,LMNA,HNRNPA1
12	Oral cavity bleeding	HP:0030140	1.23E-06	8.21E-05	NUMA1,NPM1,FIP1L1
13	Disseminated intravascular coagulation	HP:0005521	1.44E-06	8.28E-05	NUMA1,NPM1,FIP1L1
14	Chronic infection	HP:0031035	1.44E-06	8.28E-05	NUMA1,NPM1,FIP1L1
15	Alcoholism	HP:0030955	2.23E-06	0.000112	NUMA1,NPM1,FIP1L1
16	Anorexia	HP:0002039	2.11E-06	0.000113	SRSF2,NUMA1,NPM1,FIP1L1
17	Stomatitis	HP:0010280	2.55E-06	0.000121	NUMA1,NPM1,FIP1L1
18	Bone marrow hypercellularity	HP:0031020	2.90E-06	0.000129	NUMA1,NPM1,FIP1L1
19	Exertional dyspnea	HP:0002875	3.07E-06	0.00013	NUMA1,NPM1,LMNA,FIP1L1
20	Weight loss	HP:0001824	3.48E-06	0.00014	SRSF2,NUMA1,NPM1,LMNA,FIP1L1
